# Gender differences in behaviors toward acceptance of donor egg, sperm, and embryo in Northern Thai infertile couples

**DOI:** 10.5935/1518-0557.20240042

**Published:** 2024

**Authors:** Kanyapat Taechapeti, Sorawit Piriyasakmontri, Supitchaya Phatai, Tanyaporn Maraka, Usanee Sanmee

**Affiliations:** 1 Division of Reproductive Medicine, Department of Obstetrics and Gynecology, Faculty of Medicine, Chiang Mai University, Chiang Mai 50200, Thailand; 2 CMEx Fertility Center, Center of Medical Excellence, Chiang Mai University, Chiang Mai 50200, Thailand

**Keywords:** gender, behavior, donor gamete, donor embryo

## Abstract

**Objective:**

This study aimed to examine the behavior towards the acceptance of donor egg, donor sperm, and donor embryo of Northern Thai infertile couples, separated between men and women.

**Methods:**

A cross-sectional study was conducted at the CMEx Fertility Center, Chiang Mai, Thailand. The questionnaires consisted of sociodemographic questions and the acceptance of couples toward donor egg, sperm and embryo. The couples filled in the answers separately.

**Results:**

A total of 250 infertile couples were assessed. There were no differences in the acceptance rate of donor egg, sperm and embryo between the men and the women. Male acceptance rates were 25.6%, 18.8%, and 18.8%, respectively; while female acceptance rates were 24.4%, 18.4%, and 19.2%, respectively. Most couples (over 70%) concordantly rejected the donation program. Around 10% of couples had discordant answers. The concordance accepted for couples for donor egg, sperm and embryo was only 20%, 13.2%, and 14.8%. Older people and those who had been infertile for a longer period were significantly more likely to accept donation programs.

**Conclusions:**

There is no difference concerning the acceptance of donor gametes and embryo among men and women. Most participants reject the utilization of donor programs, the overall acceptance rate is relatively low. This may indicate the need for more adequate information and education for the community to enhance prevention programs rather than focus on the treatment with donor gametes or embryos.

## INTRODUCTION

Although advancements in Assisted Reproductive Technology (ART) are undeniably effective and have guaranteed an immense number of successful results, gamete quality remains the key to successful conception and child delivery. If the reproductive cells are defective or either of the couples is unable to produce the gamete, donor eggs, sperm, or embryos may have to be utilized to overcome infertility.

The trends of adopting donor gametes and embryos have been increasing. In 2019, ART using donor gametes and embryos produced over 4,100 successful births in the United Kingdom, with 1 of every 170 births attributed to donor gametes and embryos. The utilization of donor eggs has increased the rate of pregnancy from 5% to 30% in females aged 43-50 (HFEA, 2022), and the pregnancy rate may be as high as 40.6% with donor embryos (Halle, 2023). In the United States, donor gametes, donor embryos, and surrogacy contributed to 16% of all ART treatments (Kushnir *et al*., 2017). These findings suggest that the utilization of donor eggs, sperm, or embryos could be a promising solution for couples suffering from absent or poor gametes.

Despite its favorable premise and outcomes, the use of these donor gametes and embryos did not seem to be as accepted in Asia, including Thailand, as it in other parts of the world, and remains a controversial topic among infertile couples and society. As there is currently no study in this field in Thailand, we would like to examine the behavior toward the acceptance of donor egg, sperm, and embryo among Northern Thai infertile couples, separating the behaviors of men and women according to the country law that the use of donor gamete and embryo for conception must have the consent from the spouse. We aim to extract and implement these data into the planning of conception attempts and to exhibit the limitations and problems of this ART modality to provide alternative solutions to infertile couples with defective reproductive cells.

## MATERIALS AND METHODS

This cross-sectional study was carried out at the CMEx Fertility Center, Chiang Mai, Thailand from March to October 2023 with the approval of the Research Ethics Committee of the School of Medicine, Chiang Mai University. The infertile couples that visited our center received study information. Voluntarily filling in the questionnaire was considered as consent. Both male and female partners completed the questionnaire separately and anonymously.

The questionnaire comprised two parts. The first part was sociodemographic background including gender, age, duration of conception attempt, child from previous and/or current marriage, education, occupation, income per month and religion. The second part was their behaviors toward the acceptance of donor egg, sperm, and embryo, structured in “Accept” and “Reject” options. Only completely filled-out questionnaires were used for data analysis.

Data was analyzed using the Chi-square test, independent sample t-test or Mann-Whitney U test based on data type and distribution of the SPSS program version 27. *p*-value <0.05 was considered statistically significant.

## RESULTS

Two hundred and fifty infertile couples were included in the study. The sociodemographic data of the participants was separated by gender are shown in Table 1. The majority of the participants, both men and women, are between 31 and 40 years old, trying to conceive for less than five years, never had children, education are at a bachelor’s degree, run a personal business, earned more than 30,000 baths per month, and are Buddhist (Table 1).

**Table 1:** Sociodemographic data from men and women participating in the study.

Parameters	Men (n=250)	Women (n=250)
Age (years) 20-30 31-40 41-50 51-60 61-70	27 (10.8) 155 (62.0) 58 (23.2) 8 (3.2) 2 (0.8)	51 (20.4) 164 (65.6) 34 (13.6) 1 (0.4) 0 (0.0)
Duration of conception attempt (years) < 5 years ≥ 5 years	159 (63.6) 91 (36.4)	159 (63.6) 91 (36.4)
Have own children No Yes, from previous marriage Yes, from current marriage	216 (86.4) 16 (6.4) 18 (7.2)	220 (88.0) 10 (4.0) 20 (8.0)
Education Under bachelor’s degree Bachelor’s degree Higher than a bachelor’s degree	78 (31.2) 135 (54.0) 37 (14.8)	53 (21.2) 145 (58.0) 52 (20.8)
Occupation Government officer Private officer Personal business Others	78 (31.2) 44 (17.6) 96 (38.4) 32 (12.8)	74 (29.6) 50 (20.0) 89 (35.6) 37 (14.8)
Income per month (baht)^*^ ≤ 10,000 10,001-20,000 20,001-30,000 > 30,000	15 (6.0) 86 (34.4) 58 (23.2) 91 (36.4)	19 (7.6) 82 (16.4) 71 (28.4) 78 (31.2)
Religion Buddhist Christian Muslim Others	227 (90.8) 17 (6.8) 2 (0.8) 4 (1.6)	227 (90.8) 21 (8.4) 1 (0.4) 1 (0.4)

Values are frequency, n (%)

* US Dollar conversion rate was 36 baht to 1 US Dollar at the time of data collection

Regarding their behavior towards the acceptance of donor gametes and embryos, the total acceptance rates for donor egg, donor sperm, and donor embryo were 25%, 18.6%, and 19%, respectively. No differences were shown in the acceptance rate of donor egg, sperm, and embryo between men and women. Male acceptance rates were 25.6%, 18.8%, and 18.8%, respectively; while female acceptance rates were 24.4%, 18.4%, and 19.2%, respectively (Table 2).

**Table 2:** Acceptance rates of donor egg, sperm and embryo according to gender.

Parameters	Men (n=250)	Women (n=250)	p	Total (n=500)
Donor egg	64 (25.6)	61 (24.4)	0.757	125 (25.0)
Donor sperm	47 (18.8)	46 (18.4)	0.909	93 (18.6)
Donor embryo	47 (18.8)	48 (19.2)	0.910	95 (19.0)

Values are frequency, n (%)

Chi-square test

Most couples concordantly rejected donor egg, sperm, and embryo (70%, 76%, and 76.8%, respectively). Around 10% of couples were discordant regarding their answer, one partner accepted and the other rejected. The concordance of acceptance of couples for donor egg, sperm and embryo were only 20%, 13.2%, and 14.8% (Table 3).

**Table 3:** Distribution of couples according to concordance of answers.

	250 Couples		
	**Donor egg**	**Donor sperm**	**Donor embryo**
Concordance “accepts”	50 (20.0)	33 (13.2)	37 (14.8)
Concordance “rejects”	175 (70.0)	190 (76.0)	192 (76.8)
Discordant answer	25 (10.0)	27 (10.8)	21 (8.4)

Values are frequency, n (%).

Table 4 provides the association of the behavior toward the acceptance of the donation program and the sociodemographic variables including age, duration of conception attempt, infertility type, education, income, and religion. Older people were significantly more likely to accept donation programs (*p*=0.002), as were those who had been infertile for a longer period (*p*=0.001).

**Table 4:** Association of sociodemographic data and attitude towards acceptance of donor egg, sperm, and embryo.

Parameters	Accept even one method (n=139)	Reject all method (n=361)	*p*
Age (years)^*^	38.0 (33.0-41.0)	35.0 (32.0-39.0)	0.002
Duration of conception attempt (years)^*^	4.0 (2.0-7.3)	3.0 (2.0-5.0)	0.001
Have own children No Yes	120 (86.3) 19 (13.7)	316 (87.5) 45 (12.5)	0.719
Education Under bachelor’s degree Bachelor’s degree or higher	41 (29.5) 98 (70.5)	88 (24.4) 273 (75.6)	0.242
Income per month (baht)^†^ ≤ 30,000 > 30,000	91 (65.5) 48 (34.5)	240 (66.5) 121 (33.5)	0.830
Religion Buddhist Others	123 (88.5) 16 (11.5)	331 (91.7) 30 (8.3)	0.268

Values are frequency, n (%), Chi-square test, unless otherwise stated

* Median (interquartile range), Mann-Whitney U test

† US Dollar conversion rate was 36 baht to 1 US Dollar at the time of data collection

## DISCUSSION

The donation program has become the ART modality of choice for many couples whose gametes of either partner is defective in quality and/or quantity. To date, there has been no study on the behavior of Thai people towards the acceptance of donor gametes and embryos. We studied infertile people, not the general population, because these people have the most opportunity to use the donation program. We suggest that individual experience plays a role in the decision to accept donor gametes and embryo. Our data from 500 infertile people found that most participants rejected the utilization of donor gametes and embryos, and the acceptance for donor egg, sperm, and embryo was only 25%, 18.6%, and 19.1%. The number was similar to that in the survey performed in India which the acceptance rate for donor egg was 32%, donor sperm was 17.4% and donor embryo was 19.7% (Banerjee & Singla, 2018). But when compared with data from the United Kingdom, 61.3% accept donor egg and 48.4% accept donor sperm, the acceptance rate of our country is much lower (Kazem *et al*., 1995). This may be attributed to the cultural differences in the conservative beliefs of family structure, in which involving gametes or embryos from a third-party may interfere with social and biological connections within the family. This suggestion is in sync with Africans, in which a child represents everything, and procreation is rather conferred by the pregnancy, which is visible, than by the genes; therefore the acceptance of donor gametes and embryos in their country is very high at 89% (Écra *et al*., 2017).

We tried to identify sociodemographic factors that may influence behavior towards the acceptance of donor gametes and embryos. Our study found a strong association between acceptance and older age, and longer duration of infertility. This finding is consistent with many studies (Kazem *et al*., 1995; Bello *et al*., 2014), suggesting that people will accept donor programs as the last option to overcome infertility. After a long period of emotional and physical exhaustion, the donor program may be the most feasible to fulfill their families. Some studies demonstrate the influence of education (Kazem *et al*., 1995), and religion (Braverman & Corson, 1995) on the acceptance rate of donor programs, which was not found in our study.

We examined the difference in behaviors between men and women, and also assessed the concordance of the couple’s answers. According to the Thailand law, no ART procedure will be done without the spouse’s consent including the donation program. We found that gender does not affect behavior towards the acceptance of donor gametes and embryos. Both men and women equally accept donor egg, sperm, and embryo, and are more amenable to accepting donor egg than sperm and embryo. The hypothetical explanation for this inclination in the preference for donor eggs is that it enables both partners to feel involved in the childbearing and to share a connection with the child, the father would share the genetic tie; and the mother could experience pregnancy and build a maternal bond with the child, all of which are impossible to achieve in cases of donor sperm and donor embryo (Bracewell-Milnes *et al*., 2016; Eisenberg *et al*., 2010).

Discordant responses to the acceptance of donor gametes and embryos by the couples lead to an inability to access the donation program that requires consent from the spouse. Our study found discordant answers by the couples in 10% for the donor egg; 10.8% for the donor sperm and 8.4% for the donor embryo. This further reduces the number of couples accessing the donation program, limited to the concordance between accepting couples, which is only 20% for donor egg; 13.2% for donor sperm and 14.8% for donor embryo. Although this number may not represent the actual number in the real situation because this questionnaire was given at the beginning of the infertility evaluation, their behavior may change as the need for their use of donor gamete and embryo becomes more imminent. However, the responses from the representative infertile couples have shown interesting trends and could be further utilized by healthcare professionals. In our data, the behavior of Thai infertile couples toward the utilization of donor gamete and embryo is relatively low.

Although changing a person’s behavior is possible, but extremely difficult, it requires a broader change in their environment and culture (Wagner *et al*., 2020). Therefore, the most possible solution seems to be a preventive modality including primary, secondary and tertiary approaches to minimize the risk of losing reproductive ability that led to the need for donor gametes and embryos. For instance, increasing public awareness of age-related fertility decline (Habbema *et al*., 2015), giving importance to fertility advice as a part of primary health care and preconception program (Vause *et al*., 2009; Anderson *et al*., 2010), early in identifying infertility risk factors and acting immediately such as further investigation or referral to a fertility specialist (Nekuei *et al*., 2013), provide adequate information on fertility preservation issues (Rodriguez-Wallberg *et al*., 2021). This strategy can result in a higher chance of successful pregnancy whether naturally or from treatment.

In conclusion, this study reflects the behavior toward the acceptance of donor gametes and embryos of infertile couples in Thailand. There was no difference in the acceptance rate among men and women. The overall acceptance of donor gametes and embryos is relatively low. This may indicate the need for more adequate information and education for the community to enhance the prevention program rather than focus on the treatment with donor gametes or embryos.
